# Effect of Phosphoric Acid and Soluble Phosphate on the Properties of Magnesium Oxychloride Cement

**DOI:** 10.3390/ma17194828

**Published:** 2024-09-30

**Authors:** Qing Huang, Su Wang, Yongsheng Du, Zhigang Yin, Bing Chen, Jie Zhang, Weixin Zheng

**Affiliations:** 1 School of Materials Science and Engineering, Shaanxi University of Technology, Hanzhong 723001, China; 18729642961@163.com; 2 National and Local Joint Engineering Laboratory for Slag Comprehensive Utilization and Environmental Technology, Shaanxi University of Technology, Hanzhong 723000, China; 3 Lithium Resources and Lithium Materials Key Laboratory of Sichuan Province, Tianqi Lithium Corporation, Chengdu 610000, China; su.wang@tianqilithium.com (S.W.); yinzg@tianqilithium.com (Z.Y.); zhangjie@tianqilithium.com (J.Z.); 4 Key Laboratory of Comprehensive and Highly Efficient Utilization of Salt Lake Resources, Qinghai Institute of Salt Lakes, Chinese Academy of Sciences, Xining 810008, China

**Keywords:** magnesium oxychloride cement, water resistance, phosphoric acid and phosphate, setting time, compressive strength

## Abstract

This study investigates the effects of phosphoric acid (H_3_PO_4_), potassium dihydrogen phosphate (KH_2_PO_4_) and sodium dihydrogen phosphate (NaH_2_PO_4_) admixtures on the setting time, compressive strength and water resistance of magnesium oxychloride cement (MOC). MOC samples incorporating different admixtures are prepared, and their hydration products and microstructures are studied via X-ray diffraction (XRD) and scanning electron microscopy (SEM). The results indicate that the addition of H_3_PO_4_, KH_2_PO_4_ and NaH_2_PO_4_ reduces the initial and final setting times and decreases the compressive strength. However, the compressive strength of MOC is higher than 30.00 MPa with the addition of 2.0 wt.% phosphoric acid and its phosphate after 14 days of air curing. The water resistance of modified MOC slurries is significantly improved. The softening coefficient of MOC with 2.0 wt.% H_3_PO_4_ is 1.2 after 14 days of water immersion, which is 3.44 times higher than that of the neat MOC. The enhancement in water resistance is attributed to the formation of amorphous gel facilitated by H_3_PO_4_, KH_2_PO_4_ and NaH_2_PO_4_. Furthermore, the improvement in water resistance is manifested as H_3_PO_4_ > KH_2_PO_4_ > NaH_2_PO_4_.

## 1. Introduction

Magnesium oxychloride cement was invented by the French scientist, Sorrell, and is also known as Sorrell cement (abbreviated as MOC) [1,2,3]. As a special type of cement, its main hydration products at room temperature are 5Mg(OH)_2_·MgCl_2_·8H_2_O (abbreviated as 518 phase) and 3Mg(OH)_2_·MgCl_2_·8H_2_O (abbreviated as 318 phase) [4]. Compared with ordinary Portland cement, MOC is an air-hardening cementitious material [5]. It has many advantages such as early strength, high strength, fast setting time, wear resistance, corrosion resistance, fire resistance, strong bonding force, low alkalinity, etc. [6,7,8,9,10]. In addition, it has good compatibility with the environment, so it can replace artificial polymer materials in the preparation of disposable products. But its disadvantages are also obvious [2,10]. Firstly, MOC has poor water resistance when immersed in water, and its strength decreases [11]. Secondly, MOC easily absorbs moisture, returns to brine and frosts in humid environments, which affects its appearance and use. Thirdly, the MOC product is prone to warping and deformation, resulting in product scrapping [12,13]. Among these, the biggest drawback is the poor water resistance of MOC.

The hydration reaction of MOC is mainly carried out in a ternary system composed of magnesium oxide, magnesium chloride and water, and its formation can be represented by the following equation [14]:

5MgO + MgCl_2_ + 13H_2_O→5Mg(OH)_2_·MgCl_2_·8H_2_O (1)

3MgO + MgCl_2_ + 11H_2_O→3Mg(OH)_2_·MgCl_2_·8H_2_O (2)

From a thermodynamic perspective, the 518 phase is a metastable phase, and the 318 phase is a stable phase. The 518 phase tends to transform into the 318 phase. However, due to its higher strength and better water resistance, it is hoped that more 518 phases will be generated in the system [15]. As the main hydration products of MOC, the 518 and 318 phases are highly prone to water absorption and hydrolysis, as shown in the following equations [16,17]:

5Mg(OH)_2_·MgCl_2_·8H_2_O +H_2_O→Mg(OH)_2_↓ + Mg^2+^ +Cl^−^ + H_2_O (3)

3Mg(OH)_2_·MgCl_2_·8H_2_O +H_2_O→Mg(OH)_2_↓ + Mg^2+^ +Cl^−^ + H_2_O (4)

After hydrolysis, the framework structure in MOC will transform to the layered structure of Mg(OH)_2_, leading to the loose structure and resulting in a significant decrease in strength [12]. There are many voids and pores inside the structure and crystallization contact points due to the multiphase porous structure of MOC [11]. From the thermodynamic perspective, the stability is relatively low and the solubility is high at the contact point of crystallization [18]. When it encounters water, it will dissolve first, and the numerous pores provide channels for water intrusion. Therefore, these structural characteristics also determine the water resistance of MOC [19].

The poor water resistance of MOC severely limits the development of the industry. At present, adding modifiers to MOC has become the most effective method [11,12,20,21,22]. Adding modifiers ensures that the hydration product crystal phase can stably exist in water. The modifiers form a water-resistant protective film on the surface of the hydration product. Or the modifiers fill the pores in the MOC that reduce the movement channels of water molecules and hydrolysis ions. Modifiers can prevent adverse phenomena such as moisture absorption, halogenation, frost and deformation [2,19,23].

The commonly used modifiers include inorganic modifiers, organic modifiers and composite modifiers [24]. From current research, although many positive measures have been proposed and achieved good results, the problem of the poor water resistance of MOC has not been fundamentally solved [25].

To improve the water resistance of MOC, various methods have been adopted. Among them, the most effective method is the incorporation of admixtures and supplementary materials to cementitious materials [26,27,28]. It is reported that the compressive strength of MOC decreases by only 10% and 15% when 1% soluble phosphate [29] and 30% fly ash [30] were added to the MOC, respectively, after 28 days of immersion in water. After immersion in water for 28 days, the compressive strength of MOC decreased by up to 20% with only 1% phosphoric acid addition, but the improvement in water resistance was significant, facilitating a 50% increase in the strength retention coefficient [12]. Additionally, different scholars have conducted relevant research and reports on the mechanism of adding phosphoric acid and its salts to MOC to improve water resistance [31,32].

In conclusion, phosphoric acid and soluble phosphate are the main modified materials currently used to improve the water resistance of MOC [11,12,33,34]. However, the majority of studies primarily concentrate on the modification of MOC through phosphate. In contrast, there is a scarcity of research that examines and compares the effects of modification on MOC using phosphoric acid versus soluble phosphate. In this paper, in order to compare and analyze the effects of phosphoric acid and soluble phosphate on the performance of MOC, the same amount of phosphoric acid and its soluble salt were added to MOC. The influence of phosphoric acid and its soluble phosphate on the properties of MOC was analyzed.

## 2. Materials and Methods

### 2.1. Raw Materials

The light-burnt magnesia used in the experiment was obtained from Liaoning Province, China. The activity of light-burnt magnesia was tested by direct hydration [35] at 105 °C and 101.3 MPa containing 50.0 wt.% active magnesium oxide (MgO_a_). The bischofite used in this experiment was mainly composed of hygroscopic magnesium chloride hexahydrate (MgCl_2_·6H_2_O), which was provided by Jiayoumeiye Ltd. (Xining, Qinghai Province, China). The phosphoric acid (H_3_PO_4_), potassium dihydrogen phosphate (NaH_2_PO_4_) and sodium dihydrogen phosphate (KH_2_PO_4_) used in this experiment were analytically pure. The chemical composition of light-burnt magnesia is listed in Table 1.

### 2.2. Specimen Preparation

The molar ratio of active MgO to MgCl_2_ to H_2_O was fixed at 7:1:19.8 for the preparation of MOC paste. Magnesium chloride solution was prepared firstly by dissolving bischofite in water. Then, the light-burnt magnesia was added in the solution to form fresh slurry. Meanwhile, H_3_PO_4_, KH_2_PO_4_ and NaH_2_PO_4_ were added as admixtures into the slurry for the incorporation of 0.0 wt.%, 0.5 wt.%, 1.0 wt.% and 2.0 wt.% (by weight of light-burnt magnesia); the detailed quantities are presented in Table 2. The MOC samples were labeled as *n*%H_3_PO_4_/KH_2_PO_4_/NaH_2_PO_4_-W-14D, where *n*% indicated the addition of H_3_PO_4_/KH_2_PO_4_/NaH_2_PO_4_, and W indicated that the sample was soaked for curing. The mixture slurry was then cast into steel molds with the size of 20 × 20 × 20 mm. Samples were finally covered with plastic sheets to prevent evaporation and initially cured for 24 h at room temperature. Specimens were then removed from the molds and further cured for another 14 days and further immersed in water for another 14 days.

### 2.3. Specimen Analysis

Setting times were determined using the Vicat test at 20 ± 2 °C [36]. The compressive strength of different specimens was measured by a material testing machine (MTS, SANYU, SYE3000B) with a maximum force of 300 kN and a loading rate of 1.52 mm min^−1^. More than three samples with different compositions and ages were tested. The 80 µm powders were prepared by crushing the samples for X-ray diffraction (XRD) analysis. Diffraction patterns were obtained using an X’Pert Pro diffractometer (PANalytical, 2θ = 5~70°, Cu Kα radiation, λ = 0.15406 Å). The Rietveld method was then used to quantify crystal phase compositions via analysis of the diffraction patterns with Topas 4.2 software [37].

The water resistance of MOC slurries was evaluated for various periods of time after being cured in air for 14 days. The compressive strength of each specimen was then measured. The strength retention coefficient (I) of each MOC paste after different immersion ages was calculated [37].

I = R_d_/R_0_ (5)

where R_d_ and R_0_ represent the compressive strength of the MOC paste after immersion in water for d days and being cured in air for 14 days without subsequent immersion, respectively.

The micro-morphologies of MOC slurries with 14 days air curing and immersion in water for different times were characterized via scanning electron microscopy (SEM, JSM-5610LV).

## 3. Results

### 3.1. Setting Time of MOC Slurries

**Figure 1:**
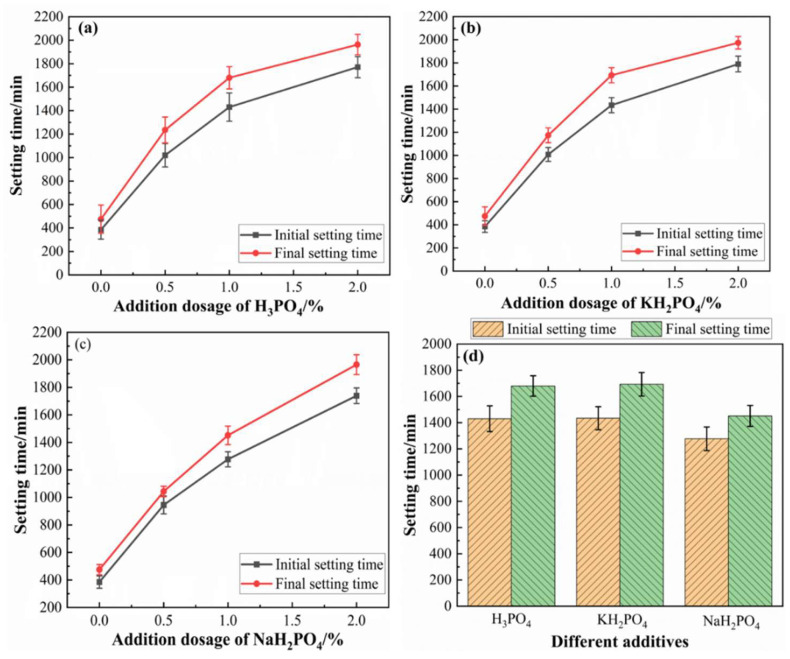
Setting time of different MOC slurries ((**a**) H_3_PO_4_, (**b**) KH_2_PO_4_, (**c**) NaH_2_PO_4_, (**d**) Setting time comparation of three MOC slurries).

The setting times of MOC slurries with different additions of H_3_PO_4_, KH_2_PO_4_ and NaH_2_PO_4_ are shown in Figure 1a–c.

Compared with neat MOC paste, the initial setting times and final setting times of MOC slurries increased with the addition of H_3_PO_4_, KH_2_PO_4_ and NaH_2_PO_4_. Meanwhile, with the increasing addition of H_3_PO_4_, KH_2_PO_4_ and NaH_2_PO_4_ from 0.0 wt.% to 2.0 wt.%, the initial setting times and final setting times of MOC slurries obviously increased. For example, the initial setting times and final setting times of MOC slurries increased by 164.94% and 160.00% with the 0.5 wt.% addition of H_3_PO_4_. The initial setting times and final setting times of MOC slurries increased by 272.47% and 256.42% with the 1.0 wt.% addition of KH_2_PO_4_. The initial setting times and final setting times of MOC slurries increased by 351.69% and 313.68% with the 2.0 wt.% addition of NaH_2_PO_4_. As a result, the addition of H_3_PO_4_, NaH_2_PO_4_ and KH_2_PO_4_ significantly prolonged the setting time of MOC.

The comparation of three types of MOC in terms of setting time is shown in Figure 1d. With the addition of 2.0 wt.% H_3_PO_4_, the initial setting time and final setting time of MOC were 1771 min and 1965 min, respectively. The initial setting time and final setting time of MOC were 1790 min and 1974 min with the addition of 2.0 wt.% KH_2_PO_4_. When adding 2.0 wt.% NaH_2_PO_4_, the initial setting time and final setting time of MOC were 1739 min and 1963 min. Among them, the addition of H_3_PO_4_ and KH_2_PO_4_ had similar effects on prolonging the setting time of MOC, while NaH_2_PO_4_ had the smallest effect on delaying the setting time of MOC. When the addition dosage was all 2.0 wt.%, the delayed effect of H_3_PO_4_, KH_2_PO_4_ and NaH_2_PO_4_ on the setting time was as follows: KH_2_PO_4_ > H_3_PO_4_ > NaH_2_PO_4_.

### 3.2. Compressive Strength of MOC Slurries

**Figure 2:**
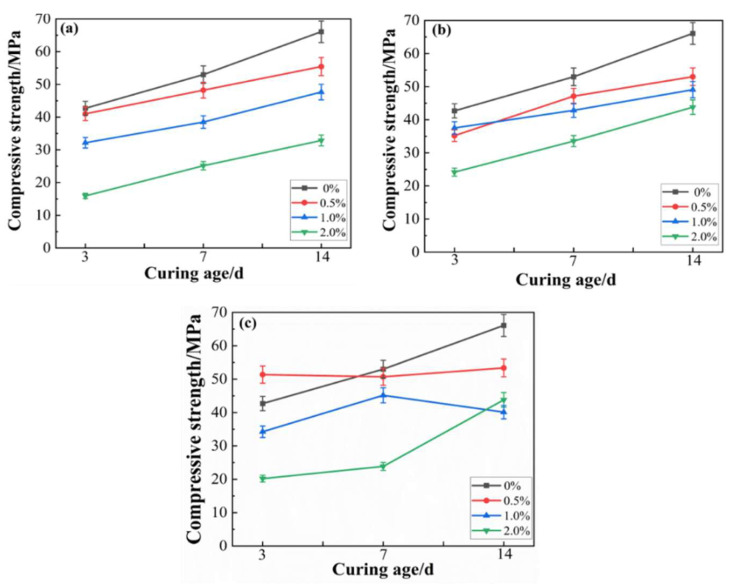
The compressive strength of MOC slurries ((**a**) H_3_PO_4_, (**b**) KH_2_PO_4_, (**c**) NaH_2_PO_4_).

The compressive strengths of MOC with different dosages of H_3_PO_4_, KH_2_PO_4_ and NaH_2_PO_4_ are shown in Figure 2a–c.

From Figure 2a, the 3-day and 14-day compressive strength of MOC samples with 0.5 wt.%, 1.0 wt.% and 2.0 wt.% H_3_PO_4_ added are 41.00 MPa, 32.16 MPa and 15.93 MPa and 55.45 MPa, 47.66 MPa and 32.86 MPa, respectively. Compared with the neat MOC sample, the compressive strength of the MOC samples with 0.5 wt.%, 1.0 wt.% and 2.0 wt.% H_3_PO_4_ addition decreased by 3.96%, 24.67% and 62.68% at 3 days, respectively. The compressive strength at 14 days decreased by 16.1%, 27.89% and 50.28%, respectively. Therefore, the addition of H_3_PO_4_ reduces the compressive strength of MOC. And the decrease in compressive strength becomes more pronounced with the increased content of H_3_PO_4_.

A shown in Figure 2b, the 3-day compressive strength of MOC samples with the KH_2_PO_4_ addition amounts of 0.5 wt.%, 1.0 wt.% and 2.0 wt.% were 51.36 MPa, 34.24 MPa and 20.20 MPa, respectively. The 14-day compressive strength was 53.37 MPa, 40.11 MPa and 43.80 MPa, respectively. Compared with the neat MOC sample, the compressive strength of the MOC samples with 0.5 wt.%, 1.0 wt.% and 2.0 wt.% KH_2_PO_4_ addition decreased by 10.31%, 19.79% and 52.68% at 3 days, respectively. The compressive strength at 14 days decreased by 19.25%, 39.31% and 33.73%, respectively. Therefore, adding KH_2_PO_4_ also reduces the compressive strength of MOC.

The compressive strengths of MOC samples with NaH_2_PO_4_ addition at a dosage of 0.5 wt.%, 1.0 wt.% and 2.0 wt.% were 41.00 MPa, 32.16 MPa and 15.93 MPa at 3 days and 55.45 MPa, 47.66 MPa and 32.86 MPa at 14 days, respectively. Compared with the neat MOC specimens, the compressive strength of the specimens with NaH_2_PO_4_ added at 0.5 wt.%, 1.0 wt.% and 2.0 wt.% decreased by 17.57%, 12.11% and 43.38%, respectively, at 3 days. The compressive strength at 14 days decreased by 19.81%, 25.72% and 33.73%, respectively. Therefore, adding NaH_2_PO_4_ also reduces the compressive strength of MOC.

As a result, the addition of H_3_PO_4_, KH_2_PO_4_ and NaH_2_PO_4_ obviously decreases the compressive strength. Additionally, the decreasing effect of H_3_PO_4_, KH_2_PO_4_ and NaH_2_PO_4_ on the compressive strength is more serious with the increasing dosage. However, the compressive strength of MOC was higher than 30.00 MPa with the addition of 2.0 wt.% phosphoric acid and its phosphate after 14 days of air curing.

### 3.3. Water Resistance of MOC Slurries

**Figure 3:**
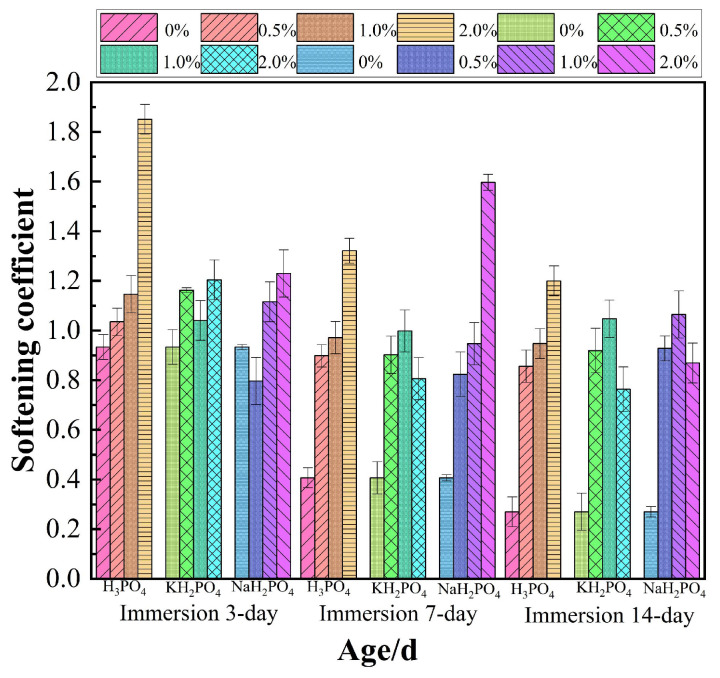
The softening coefficient of MOC slurries.

The softening coefficients of MOC slurries with different dosages of H_3_PO_4_, KH_2_PO_4_ and NaH_2_PO_4_ are shown in Figure 3 at different immersion ages. It is clear to see that the softening coefficients of MOC slurries were all higher than neat MOC after the incorporation of H_3_PO_4_, KH_2_PO_4_ and NaH_2_PO_4_. That is to say that the addition of H_3_PO_4_, KH_2_PO_4_ and NaH_2_PO_4_ can enhance the water resistance of MOC.

**Figure 4:**
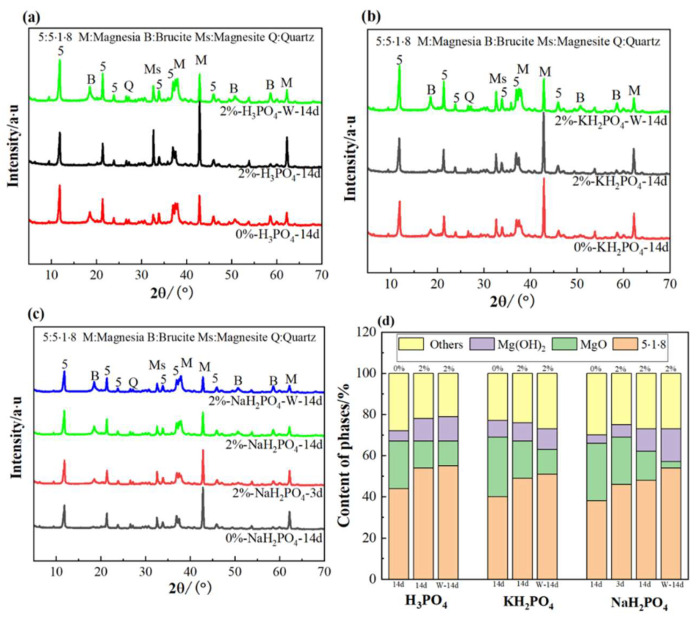
XRD patterns and crystalline phase contents of different MOC slurries cured in air for 14 days ((**a**) H_3_PO_4_, (**b**) KH_2_PO_4_, (**c**) NaH_2_PO_4_, (**d**) The crystalline phase contents of three MOC slurries).

As can be seen from Figure 4, the addition of 1.0 wt.% H_3_PO_4_ obviously improved the water resistance of MOC after 14 immersion days. The softening coefficient of MOC with 2.0 wt.% H_3_PO_4_ was 1.2 after water immersion for 14 days, which was 3.44 times higher than the neat MOC. Additionally, we can clearly see that the optimal dosage of H_3_PO_4_ is 2.0 wt.% for the MOC.

As illustrated in Figure 4, the softening coefficients of MOC samples incorporated with KH_2_PO_4_ and NaH_2_PO_4_ after a 14-day immersion period are comparable. Furthermore, their softening coefficients exhibited a trend of initially increasing followed by decreasing as the inclusion amount rose. The optimal concentrations of KH_2_PO_4_ and NaH_2_PO_4_ were determined to be 1%. Through the comparation, it was found that the enhancement effects of H_3_PO_4_, KH_2_PO_4_ and NaH_2_PO_4_ on the softening coefficient of MOC were different. Overall, adding H_3_PO_4_ had the best effect on the softening coefficient. The water resistance manifested as H_3_PO_4_ > KH_2_PO_4_ > NaH_2_PO_4_.

### 3.4. XRD Patterns

The XRD patterns of MOC with H_3_PO_4_, KH_2_PO_4_ and NaH_2_PO_4_ cured in air for 14 days are shown in Figure 4a–c, and the corresponding quantitative mineralogical compositions are presented in Figure 4d.

The primary crystallized hydration product present in all of the MOC specimens was the 518 phase, regardless of the incorporation of H_3_PO_4_, KH_2_PO_4_ or NaH_2_PO_4_. The 518 phase contributes predominantly to the mechanical strength of MOC [37,38,39]. In addition, compared with the neat MOC sample, the content of the 518 phase in the MOC specimens with the addition of H_3_PO_4_, KH_2_PO_4_ and NaH_2_PO_4_ was relatively low. This proves that the addition of H_3_PO_4_, KH_2_PO_4_ and NaH_2_PO_4_ can reduce the early mechanical strength of MOC. However, the content of the 518 phase significantly increased after 14 days of immersion in water, indicating a significant improvement in its water resistance.

### 3.5. SEM Images

**Figure 5:**
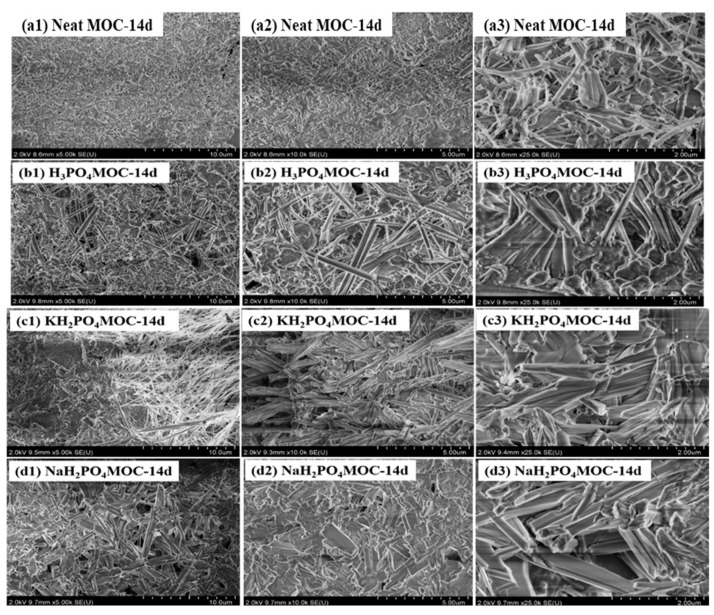
SEM images of MOC air cured for 14 days with H_3_PO_4_, KH_2_PO_4_ and NaH_2_PO_4_.

The microstructure morphology has a direct impact on the mechanical properties of MOC slurries. Figure 5 shows SEM images of neat MOC, MOC with 2.0 wt.%. H_3_PO_4_, MOC with 2.0 wt.% KH_2_PO_4_ and MOC with 2.0 wt.% NaH_2_PO_4_ after 14 days of air curing. From Figure 5a, it can be seen that the 518 phase in the MOC without admixtures was covered with slender rod-shaped crystals, with clear edges and coarse shapes in structure. The rod-shaped crystals interlaced and overlapped with each other, resulting in the higher strength of the MOC. After modification with H_3_PO_4_, the 518 crystals transformed into block and flake shapes, with wide and smooth shapes and blurred edges. After modification with KH_2_PO_4_, the 518 crystals of the MOC changed to sheet-like shapes, with wide and smooth shapes and gradually blurred edges. After modification with KH_2_PO_4_, the 518 crystals in the MOC also became large and smooth. However, compared with the H_3_PO_4_ MOC and KH_2_PO_4_ MOC, the 518 phase crystals of the KH_2_PO_4_ MOC had relatively clear edges and lower edge blurring.

**Figure 6:**
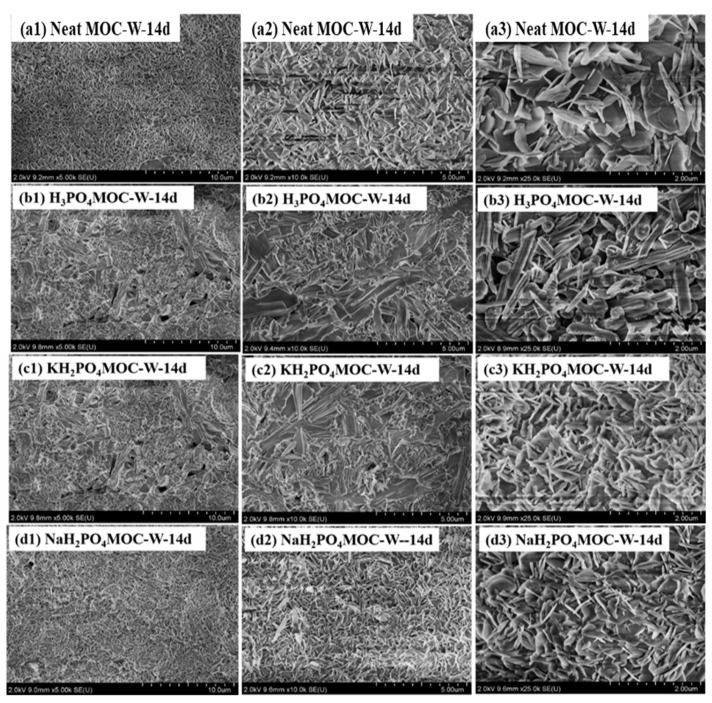
SEM images of MOC immersed in water for 14 days with H_3_PO_4_, KH_2_PO_4_ and NaH_2_PO_4_.

Figure 6 shows the SEM images of neat MOC, 2.0 wt.% H_3_PO_4_ MOC, 2.0 wt.% KH_2_PO_4_ MOC and 2.0 wt.% NaH_2_PO_4_ MOC soaked in water for 14 days. From Figure 6a, it can be seen that after being soaked in water for 14 days, the surface of the matrix was mainly composed of a large number of leaf shaped crystals, with loose and porous structures. Water invaded the interior of the cement along the pores, decomposing the 518 phase into Mg(OH)_2_ and MgCl_2_, resulting in poor mechanical properties of MOC after immersion [29,30], and showing poor water resistance. In Figure 6b, it is found that the matrix surface of MOC with H_3_PO_4_ added was mainly composed of a large number of sharp rod-shaped crystals after being soaked in water for 14 days. The rod-shaped crystals overlapped and crosslinked with each other, indicating that the addition of H_3_PO_4_ changes the morphology of MOC. By comparing Figure 5 and Figure 6, we can observe the microstructure changes in MOC samples before and after immersion with H_3_PO_4_, KH_2_PO_4_ and NaH_2_PO_4_. It can be seen that the structural morphology of MOC underwent certain changes after immersion. But the structure mainly contained rod-shaped crystals. It can be observed that amorphous substances attached to the crystals on the surface and filled some gaps between the crystals, thus preventing the contact between internal crystals and water. This can thereby improve the water resistance of MOC.

## 4. Discussion

On the whole, the addition of H_3_PO_4_, KH_2_PO_4_ and NaH_2_PO_4_ prolonged both the initial and final setting time of MOC (Figure 1). The acid group of H_3_PO_4_, KH_2_PO_4_ and NaH_2_PO_4_ can absorb on the surface of MgO particles to form Mg_3_(PO_4_)_2_. This further restricts the hydration of MgO particles. H_3_PO_4_, KH_2_PO_4_ and NaH_2_PO_4_ also form coordination bonds with Mg^2+^ in phase 5, which delays the hydration and setting time of MOC cement. Although H_3_PO_4_, KH_2_PO_4_ and NaH_2_PO_4_ delay hydration, the hydration products are similar. XRD data show that phase 518 was formed in the cement formed by H_3_PO_4_, KH_2_PO_4_ and NaH_2_PO_4_. The retarded setting time match the results in the literature [40,41].

H_3_PO_4_, KH_2_PO_4_ and NaH_2_PO_4_ reduce the initial and later strength of MOC. Other researchers also have found that additives reduce the strength of MOC before 28 days [10,32]. The reason for the decreased compressive strength is the slowing down of the hydration rater of MOC. Additionally, the content of 518 phase in MOC specimens with the addition of H_3_PO_4_, KH_2_PO_4_ and NaH_2_PO_4_ was relatively low, which proves the decrease in compressive strength. The compressive strength of MOC is directly related to its microstructure. And the contribution of blocky and flaky crystals to strength is much smaller than that of fibrous and needle-like crystals. Therefore, the addition of H_3_PO_4_, KH_2_PO_4_ and NaH_2_PO_4_ significantly reduced the early compressive strength of MOC. The reason for this is that the addition of H_3_PO_4_, KH_2_PO_4_ or NaH_2_PO_4_ provides a certain amount of [PO_4_]^3−^. The addition of [PO_4_]^3−^ can transform the 518 crystals into low-crystallinity gel crystals, thereby decreasing the compressive strength of MOC [12].

The incorporation of phosphoric acid and phosphate into the MOC system demonstrated enhanced water resistance, likely attributable to the ionization reactions of phosphoric acid and phosphate within the MOC matrix. The specific ionization reaction is as follows:

H_3_PO_4_→H^+^ +H_2_PO_4_^−^ (6)

H_2_PO_4_^−^→H^+^ +HPO_4_^2−^ (7)

HPO_4_^2−^→H^+^ +PO_4_^3−^ (8)

Magnesium oxide undergoes a hydration reaction when exposed to water. The specific hydration reaction is as follows:

MgO + H_2_O→Mg(OH)_2_ (9)

Mg(OH)_2_→Mg^2+^ +2OH^−^ (10)

The H^+^ generated from Equations (6)–(8) undergoes a neutralization reaction with the OH^−^ generated from Equation (10), as shown in Equation (11). The resulting PO_4_^3−^ and Mg^2+^ generated from Equation (10) form a magnesium phosphate phase, as shown in Equation (12) [39]:

H^+^ +OH^−^→H_2_O (11)

2PO_4_^3−^ + 3Mg^2+^→Mg_3_(PO_4_)_2_ (12)

Due to the fact that the ionization equilibrium constant of HPO_4_^2−^ is smaller than that of H_2_PO_4_^−^, when the initial molar concentration is the same, the H^+^ concentrations of H_3_PO_4_, KH_2_PO_4_ and NaH_2_PO_4_ in MOC are H_3_PO_4_ > KH_2_PO_4_ > NaH_2_PO_4_. KH_2_PO_4_ and NaH_2_PO_4_ are both strong electrolytes that are highly soluble in water. And the stability of K^+^ and Na^+^ is K ^+^ >Na^+^.

Research [33] shows that after immersion in H_3_PO_4_ solution, the 518 phase crystals are significantly reduced, and a large amount of phosphate (MgHPO_4_·3H_2_O) is generated. The generated MgHPO_4_·3H_2_O is slightly soluble in water, and its encapsulation on the surface of the sample can block the contact between water and 518 phase crystals, effectively preventing 518 phase hydrolysis and improving water resistance. Therefore, the phosphate generated by H_3_PO_4_ and Mg^2+^ in cement paste can be the main reason behind the prevention of the hydrolysis of 518 crystals. The magnesium phosphate formed by it covers the surface of the crystals, which can effectively block the invasion of water and improve the water resistance of MOC. Due to the fast setting and hardening rate of MOC, it is difficult for [PO_4_]^3-^ and Mg^2+^ to migrate on the surface of the hydration product crystal phase, and it is difficult for a crystal phase to form with phosphate. The surface of the hydration product can be amorphous and irregularly distributed. In addition, the amount of H_3_PO_4_, KH_2_PO_4_ and NaH_2_PO_4_ is very small, so magnesium phosphate cannot be detected by XRD and other methods when added internally. In addition, after soaking in water for 14 days, the content of 518 phase increased relatively. Another reason for this may be that under this experimental design ratio, there was an excess of active magnesium oxide. When soaked in water, secondary hydration occurred, resulting in an increasing trend of 518 phase content in MOC.

Compared with the neat specimen, the addition of H_3_PO_4_, KH_2_PO_4_ and NaH_2_PO_4_ changed the morphology of the main crystal 518 in MOC. Before soaking in water, the 518 phases in neat MOC appeared to have columnar shapes with clear edges and were interlaced with each other, exhibiting great compressive strength. After adding H_3_PO_4_, KH_2_PO_4_ and NaH_2_PO_4_ to MOC, the 518 crystals began to shrink in shape. It is suspected that this may be due to the hydrolysis of H_3_PO_4_, KH_2_PO_4_ and NaH_2_PO_4_, which provides [PO_4_]^3−^ and reacts with Mg^2+^ in MOC to generate amorphous magnesium phosphate that covers the surface of 518 crystals. As magnesium phosphate salts are insoluble substances, their solubility is much lower than that of 518 crystals. This contributes to the good water resistance of MOC.

## 5. Conclusions

In this paper, to compare the addition of H_3_PO_4_, KH_2_PO_4_ and NaH_2_PO_4_ as additives on MOC, the effects on the setting time, compressive strength, water resistance, phase composition and microscopic composition of MOC are examined. The main conclusions are as follows:

1. The addition of H_3_PO_4_, KH_2_PO_4_ and NaH_2_PO_4_ has a serious retarding effect on the initial setting time and final setting time of MOC. The delayed effect of H_3_PO_4_, KH_2_PO_4_ and NaH_2_PO_4_ on the setting time is as follows: KH_2_PO_4_ > H_3_PO_4_ > NaH_2_PO_4_.
2. The addition of H_3_PO_4_, KH_2_PO_4_ and NaH_2_PO_4_ obviously decreases the compressive strength. The decreasing effect of H_3_PO_4_, KH_2_PO_4_ and NaH_2_PO_4_ on the compressive strength is more serious with the increasing dosage. However, the compressive strength of MOC is higher than 30.00 MPa with the addition of 2.0 wt.% phosphoric acid and its phosphate after 14 days of air curing.
3. The addition of H_3_PO_4_, KH_2_PO_4_ and NaH_2_PO_4_ can enhance the water resistance of MOC. The optimal dosage of H_3_PO_4_ is 2.0 wt.% for the MOC, and the optimal concentrations of KH_2_PO_4_ and NaH_2_PO_4_ are determined to be 1%. The water resistance is manifested as H_3_PO_4_ > KH_2_PO_4_ > NaH_2_PO_4_.
4. The primary crystallized hydration product present in all of the MOC specimens is the 518 phase, regardless of the incorporation of H_3_PO_4_, KH_2_PO_4_ and NaH_2_PO_4_.
5. The addition of H_3_PO_4_, KH_2_PO_4_ and NaH_2_PO_4_ can transform the 518 crystals into low-crystallinity gel crystals, thereby improving the water resistance of MOC.

## Figures and Tables

**Table 1:** Chemical composition of light-burnt magnesia.

Component	MgO	MgO_a_	CaO	Al_2_O_3_	SiO_2_	Fe_2_O_3_	LOI
Mass fraction/wt.%	84.82	42.22	1.60	1.58	6.01	0.65	5.44

**Table 2:** The amount of modifier used in MOC.

Label of MOC	H_3_PO_4_	KH_2_PO_4_	NaH_2_PO_4_
W-14D	0	0	0
*n*% H_3_PO_4_-W-14D	0.5	0	0
	1.0	0	0
	2.0	0	0
*n*% KH_2_PO_4_-W-14D	0	0.5	0
	0	1.0	0
	0	2.0	0
*n*% NaH_2_PO_4_-W-14D	0	0	0.5
	0	0	1.0
	0	0	2.0
